# Profiling Collapsing Half Marathon Runners—Emerging Risk Factors: Results from Gothenburg Half Marathon

**DOI:** 10.3390/sports8010002

**Published:** 2019-12-25

**Authors:** Amir Khorram-Manesh, Therese Löf, Mats Börjesson, Finn Nilson, Sofia Thorsson, Fredrik Lindberg, Eric Carlström

**Affiliations:** 1 Department of Surgery, Institute of Clinical Sciences, Sahlgrenska Academy, University of Gothenburg, 413 45 Gothenburg, Sweden; 2 Research and Development Unit, Swedish Armed Forces Defense Medicine Centre, 426 05 Gothenburg, Sweden; 3 Sahlgrenska Academy, University of Gothenburg, 405 30 Gothenburg, Sweden; 4 Department of Neuroscience and Physiology, Sahlgrenska Academy, University of Gothenburg, 405 30 Gothenburg, Sweden; 5 Department of Food, Nutrition and Sport Science, Sahlgrenska University Hospital, 405 30 Gothenburg, Sweden; 6 Department of Environmental and Life Sciences, Karlstad University, 651 88 Karlstad, Sweden; 7 Department of Earth Sciences, University of Gothenburg, 405 30 Gothenburg, Sweden; 8 Institute of Health and Care Sciences, Sahlgrenska Academy, University of Gothenburg, 405 30 Gothenburg, Sweden; 9 School of Business, University of Southeast Norway, 3679 Notodden, Norway

**Keywords:** endurance, health, medicine, psychology, collapsing, pre-race test

## Abstract

Among several serious medical conditions, arrhythmia and heat stroke are two important causes of death during endurance races. Clinically, collapsing might be the first sign of these serious conditions and may mimic the more common and benign exercise-associated collapse. Several risk factors have been reported in the literature. We aimed to conduct a qualitative study to find a perceived risk profile among runners who collapsed and who were transported by ambulances to the nearest hospital during Gothenburg’s half marathon (2010–2017). Collapsing runners seem to lack the ability to make a decision to withdraw from the contest despite being exhausted. They feel the pain, but are unable to put meaning to their feeling, to adjust their pacing, and to handle other influences. Consequently, they do not overcome the problem or assess the situation. These individual mental characteristics may indicate a unique profile for collapsing runners. Pre-race health control and educational initiatives aiming at mental preparedness and information before endurance races might be a necessary step to avoid life-threatening complications.

## 1. Introduction

Although most people begin running to improve their physical fitness, personal challenges have proven to be the main reason for taking part in a running contest [1,2]. Running a half or full marathon can be enjoyable and raises the feeling of self-satisfaction; however, it may also contribute to medical encounters with adverse outcomes [2,3,4,5]. Among many serious and life-threatening medical complications such as ischemic heart disease; serious metabolic complications; serious heat-related disorders; and serious fluid, electrolyte or acid–base abnormalities, arrhythmia and heat stroke are two less common causes of death during endurance races [3,4,5]. Clinically, collapsing might be the first sign of these serious conditions and may mimic the more common and benign exercise-associated collapse (EAC) [3,5], which is believed to be the result of a combination of postural hypotension and blunted reflex activity [6]. Several individual or environmental factors can influence EAC.

### 1.1. Individual Factors

Running technique or pacing is the ability of the athletes to distribute their energy equally throughout the long-distance race and can vary depending on the individual [7,8]. Previous studies have shown that this ability is more developed among the experienced athletes, who can also adjust their energy use before their body temperature rises to high levels [8,9,10]. Many factors such as knowledge about the length of the race, ambient air temperature, and topography can influence runners’ pacing and consequently the energy distribution. Wrong pacing can result in a collapse [10,11]. Another component of the running technique is training load. Previous studies have shown that at least 30 km training load per week before a marathon race decreases the risk for injuries [11]. There is, however, no information about the amount of training load needed to avoid a collapse.

Health conditions such as chronic diseases and viral infections influence the individual runner’s ability to complete a race [12,13,14,15]. Coxsackievirus with cold symptoms can result in myocarditis [16,17]. Rhinovirus influences the runner’s step, making the steps longer and slower and probably increases the risks for injuries, but it has not proven to affect the lung capacity and runners’ performance [12,13,14,15].

Several publications emphasize the importance of nutrition and carbohydrates intake days before endurance races and an extra load 2–4 h before the race to increase the performance [18,19,20,21]. Fluids, on the other hand, are necessary to compensate for the losses due to perspiration [19,21,22,23,24]. Many factors may influence the perspiration mechanism such as genetic predisposition, the ability to adjust to the heat, the intensity of physical activity, and the temperature itself [20,23]. Some studies recommend substitution of up to 50–80% of the total loss due to sweat [20]. However, intake of a more considerable amount of fluids may also have side effects such as gastrointestinal issues, and hypo- or hypernatremia [23,24].

### 1.2. Environmental Factors

Recent studies from Gothenburg show a significant correlation between air temperature and number of collapses, number of ambulance transportations due to collapses, and the number of runners who could not finish the race (Non-finishers = NF). These studies used a “physiologic equivalent temperature (PET)” index to estimate the impact of heat [25,26,27]. The PET index models the thermal conditions of the human body in a physiologically relevant way and includes all meteorological variables relevant for heat exchange between the body and its environment, i.e., air temperature, wind speed, atmospheric pressure, and mean radiant temperature. The latter describes the environmental radiative load on a person and is one of the essential meteorological variables governing the human energy balance and thermal comfort [25,26,27,28]. An increase in PET of one degree is correlated with an increase in the number of collapses of 1.8 times and the number of NF with 66% [25]. Another recent study indicated the possibility of predicting the location of collapses, based on the grade of exhaustion experienced by runners. The localization of ambulance pickups was correlated with the areas where runners experienced maximal exhaustion and collapsed [29].

Although several partly unexplained risk factors have been associated with EAC, qualitative studies dealing with this issue are scarce. Defining a runner profile or identifying specific risk factors may prevent medical encounters, and open up new research fields, thus contributing to a better understanding of collapses during endurance races; furthermore, it can enable future pre-race tests and education. We aimed to conduct a qualitative study to find a perceived risk profile among runners who collapsed and who were transported by ambulance to the nearest hospital during Gothenburg’s half marathon (2010–2017).

## 2. Materials and Methods

### 2.1. General Information

Gothenburg’s half marathon (Göteborgsvarvet = GV), is the world’s largest half marathon with over 45,000 competitors [29]. Although the number of participants may vary, around 450 to 2250 medical encounters can be expected during the contest, with the variation strongly dependent on weather-related factors. Most of these medical encounters (>90%) are moderate and can be treated on-site by the organizer’s medical crew. Around 59–85% of these cases are EACs, which typically occur once the athlete stops running. However, 2–4% of all medical cases requires ambulance transportation and care at the hospitals due to their serious nature [30,31].

### 2.2. Participants from Gothenburg Half-Marathon 2010–2017

The Gothenburg Ambulance Journal was used to identify all runners picked up by ambulance due to collapse (n = 164) [32]. Runners with incomplete journals, wrong address, or information were excluded (n = 80). Remaining 84 received the template by post, but only 30 answered. Of 54 who did not reply, 40% were unknown to the local post office, 40% have moved to other areas without leaving a forwarding address, and in 20%, no reason was found. Two out of 30 individuals were excluded later since their participation was confirmed to be before the study period. The remaining 28 were included and analyzed in this study. The results of the questionnaire were completed with semi-structured telephone interviews. Of 28 participants, 15 were excluded due to sickness or unwillingness to participate in the interview. Remaining 13 participants were interviewed.

### 2.3. Review and Development of the Questionnaire

A questionnaire was developed using the following keywords in a literature study: marathon, half marathon, risk factors, medical encounters, collapses, runners’ profiles, requiring ambulance. A group of multidisciplinary professionals (n = 7) with medical (sports medicine, surgery, prehospital care and physiotherapist) and non-medical backgrounds (urban climate, human thermal comfort, and crowd management) evaluated and edited the questionnaire. Free comments were allowed and categorized under the main topics. The final version of the questionnaire was approved and was sent out to all included participants. It included the following parts (Supplementary material):

- General information: age, gender, education, profession.
- Preparation: Number of earlier contests. Average km ran in one year and 3–4 weeks before the actual contest. Factors negatively influencing the actual contest. Feeling over-confidence. Studying the map and test running the track before the contest. Checking the weather and comfortable temperature to run. Optimal dress and the color of choice. Aimed finishing time. Food and drink taken before the contest.
- Health information: Any actual diseases. Any disease in the family. ADHD diagnostic criteria for adults [33]. Collapse during physical activity. Physical condition last three weeks before the contest. Alcohol intake (number of occasions and glasses).
- Contest: Planned vs. actual finishing time. Intake of water before the collapse.
- Incident: Marking on the map (incident, ambulance pickup, when it started to be tough). Three critical factors influencing the collapse. Intrinsic factors and experiencing symptoms before collapse (stress, inner demand, nervousness, hunger, thirst, tiredness, cramps, dizziness, confusion, nausea, visual disorder, headache, strange thoughts, others). Extrinsic factors experienced before the collapse (public behavior, crowding, relatives, public cheers, uphill slope, uneven track, warmth, cold, upwind, rain, other). Potential measures to prevent collapsing.
- Post-incident: Any help while waiting for ambulance. Hospital admission and sick leave. Running after the incident and new contests. Other comments?
- Future contact: Available for new contact by email or mobile number.

### 2.4. Interviews 

In order to compensate for potential shortcomings in the questionnaire, to evaluate the response given by each participant, and to obtain spoken accounts, each responding runner was asked to take part in an interview. Exclusion criteria for interviews were (1) severe ongoing disease (defined as a medical condition that does not allow the patients to participate in an interview (one person with psychological disorder, and one with brain damage after cardiac arrest)); or (2) refusal to participate. The obtained information was transcribed and analyzed verbatim. The process of analysis was based on systematic text condensation and divided into two parts: analysis of the incident and analysis of influencing factors [34].

### 2.5. Ethical Approval 

The ethical committee approved the study (No. 177-16). Each participant received information regarding the study, its aims, and the use of data upon participation. All participants signed a letter of consent before the interviews. They also received verbal information. In both cases, they were informed that their participation was voluntary and withdrawal from the study was possible whenever they desired.

## 3. Results

### 3.1. The Results of the Questionnaire 

#### 3.1.1. Personal Information

**Figure 1:**
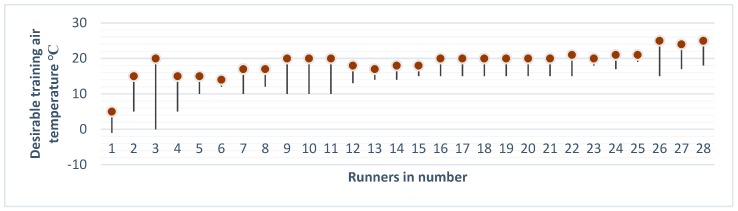
Convenient training air temperature for each runner (n = 28).

- The average age of the respondents was 41 years (n = 28, 71% men and 29% women). Around 60% had an undergraduate/graduate degree from a university (average in the Swedish population, 42%).
- The participants reported an air temperature between 12 °C and 19 °C as the most comfortable temperature for running (average = 15.6 °C, SD = 4.1 °C). (Figure 1).
- Sixteen runners (57%) reported no previous diseases, while four had pulmonary diseases, including asthma. One suffered from Reynaud’s disease; two runners had high blood pressure; two were allergic; one had diabetes; one cardiovascular disease; one suffered from depression; and one runner reported kidney disease and hypotension.
- Ten participants (36%) reported previous collapses during contests and one during exercises.
- Twelve runners (43%) reported hereditary diseases, of whom five reported more than one disease in their family (Table 1).

#### 3.1.2. Preparation before the Contest

- Nineteen of 28 participants (68%) had taken part in GV or equivalent races in the last five years before the collapse (Ran 1 to 17 times, an average of 4.2 + 3.9 times).
- Eighteen runners (64%) had run GV before, of whom 15 could report their running time (ran 80 to 141 min, average 107 + 18 min). None of the remaining 10 test ran the track before the contest.
- The respondents prepared themselves with running 19.7 km on average per week, within 12 months before the actual contest, with an increase to an average of 26.7 km 3–4 weeks pre-contest.
- Thirteen of 28 runners (46%) reported one or two factors with a possible negative impact on their result within one month before the contest. These factors were: (1) infections/flu; (2) stress/high workload; (3) sleeping problem the week before the collapse; (4) change of weather/high temperature; (5) chronic disease (depression and arrhythmia); (6) festivities and (7) pain.
- Eleven of 28 participants (39%) reported being over-confident in themselves and their performance.
- Nineteen participants (68%) did not study the race map before the start.
- Nineteen runners (68%) checked the weather report, days to hours before the contest.
- Only two (7%) respondents believed that they did not have an optimal running outfit. One reported being overdressed and the other had black clothes. The remaining respondents who reported optimal dressing had a different mix of light and dark colors.
- Regarding nutrition, the amount of the food and the time it was taken varied from a sandwich to a complete breakfast a few hours to minutes before the contest. Some did not have breakfast, and some had only lunch. Others had no lunch but breakfast. Some drank water, tea, or coffee, and others had energy drinks.

#### 3.1.3. During the Contest

- Twenty-five runners (89%) predetermined their running time, while two did not. One runner did not reply to the question. Seventeen runners (61%) ran as they planned, while nine reported slower speeds, and three faster speeds.
- Twenty-two runners (79%) reported intake of fluid on predesignated water stations. Four did not take water, and two did not reply.
- The most common intrinsic factors before collapsing were symptom/feelings experienced by runners: tiredness (60.7%), followed by dizziness (50%), and intense inner demands (42.9%). Six runners (21%) experienced all these three symptoms/feelings, and 10 of 28 runners (35.7%) experienced tiredness and dizziness in combination. Other essential symptoms were: nausea, confusion, visual disorder, thirst, cramps, nervousness and headache.
- The most common extrinsic factor before collapsing was high temperature, followed by uphill slope, public cheers, public behavior, narrow track, crowding, and relative bystanders.
- Twenty-two runners believed they could prevent their collapses, while two could not, and two were not sure. Two runners did not reply. The following measures were perceived as preventive to avoid a collapse: Slower tempo/do not put pressure on yourself/Have fewer demands on yourself/Listen to your body; Do not run at all/Stop running; Take in more fluids; Eat more; Take more salt; Exercise more; Test the track before; Wear less clothes and Exercise in high temperature.
- Six runners (21%) did not remember who helped them after collapsing, while the remaining participants mentioned organizers, healthcare staff and spectators.

#### 3.1.4. Post-Collapsing Period 

Six of 28 runners (21%) were transported and hospitalized. Two runners stayed one night and three stayed two nights at the hospital. Four of these runners had a sick leave of 2 to 3 days. One 30-year old runner suffered a cardiac arrest and brain injury and was treated at the intensive care unit followed by long-term neuro-rehabilitation. Two runners did not answer the question.

### 3.2. The Results of the Interviews 

**Figure 2:**
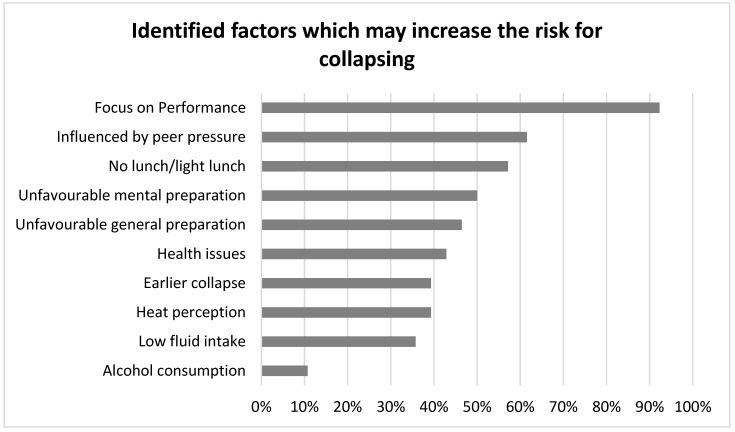
Shows most important factors influencing collapses, based on the questionnaire and interviews.

The interviews aimed to get a broader insight into some of the statements from the questionnaire. The runners had the opportunity to expand their narratives and report more in detail about their experiences. Each interview took around 40 min. The results were categorized as: the incident, runners’ characteristics and the perceived influencing factors leading to collapses (Figure 2). Thirteen runners were included in the interviews, whose age, gender, and education were comparable with the larger population who replied to our questionnaire. 

#### Incident and Individual Characteristics

- Three of the interviewees collapsed more than once during the study period, but only on one occasion did they need ambulance transportation to the hospital. Two of interviewees collapsed long after finishing the race and while on public transportation, but still needed ambulances. In total, these 13 runners suffered 16 collapses of which 11 took place during the years with the highest air temperature and highest PET.
- All interviewees exercised regularly 3.4 h per week before the contest. The most common type of exercise was running; however, rarely over 15 km per training session.
- Most of the participants had an extra intake of carbohydrates weeks before the contest and until the contest day. Most participants had a large breakfast, but ten runners skipped the lunch. Most of those reporting less intake of food could not schedule their nutritional intake on time. Three runners did not eat due to being nervous. Five runners had proper fluid intake before the contest but did not drink during the contest. Others reported sufficient control over their fluid intake.
- Few runners experienced immense stress to get to the race on time. One person could not sleep but still took part in the race since he believed that he was experienced enough to handle that.
- Besides medical conditions mentioned in the questionnaire, some runners claimed to have orthopaedic injuries before the contest (included in the health issues, Figure 2).
- Despite the collapses, few runners reported previous collapses as a favorable factor, contributing to useful knowledge and understanding of their body and a better ability to pace themselves.
- All interviewees described themselves as stubborn, ambitious and disciplined persons. Most of them measured their performance based on figures such as the number of kilometers, pulse, or time. They emphasized that these characteristics were the primary motivation for training, but even possibly the major reason for the collapse. Some reported the importance of pacing and ability to adjust their performance based on the actual circumstances. However, sometimes, the inner demands, pressure from other runners, and the comparison of last year results to the actual contest influenced their running behavior, and they continued running even though their body signaled exhaustion.
- The most remarkable post-contest experience for many of the runners was the inability to remember crucial details such as names, numbers, addresses, etc. after the event. Some believed they had a nervous breakdown and cerebrovascular insult. Many had experienced fear and anxiety, and many emphasized that the event had had an impact on their lives and training routines.

## 4. Discussion

We aimed to conduct a qualitative study to find a perceived risk profile among runners, who collapsed, and who were transported by ambulance to the nearest hospital during Gothenburg’s half marathon (2010–2017). The most important influencing factors found in this study were mental status and the high demands on individual performance placed on each collapsed runner. Another result was the number of participants (36%) who had experienced collapses during running contests before the study and the number of participants (43%) who reported hereditary issues.

Runners’ tendency to collapse might indicate other mechanisms behind collapses than diseases and physical conditions. The interviewed participants claimed they continued the contest although their body signaled exhaustion. Although most of the runners who collapsed in our study were amateurs, they all had experiences of running contests. Most of them were well educated and highly confident about their capabilities. This population is similar to another study documenting the characteristics of 161 km ultra-marathoners, in which participants were mostly well- educated, middle-aged, married men who rarely missed work due to illness or injury, generally used vitamins and or supplements, and maintained appropriate body mass with ageing [35]. The participants described themselves similarly as stubborn, ambitious, disciplined and performance-oriented.

In another published study from 2016, ten runners volunteered to describe their experiences of withdrawal during an ultra-trail race underwent interviews to share common individual characteristics leading to withdrawal. Seven representative sequences were identified: feeling pain; putting meaning to those feelings; adjusting one’s running style; attempting to overcome the problem; other runners’ influences; assessing the situation; and deciding to withdraw [36]. Consequently, our results, as well as those represented in Hoffman’s reports, may indicate that runners with specific characteristics may lack the control mechanism to maintain these representative sequences. Thus, they feel the pain and have symptoms such as tiredness and dizziness, but cannot put meaning to the feeling, and have difficulties to adjust their running style (pacing), while trying to overcome their problems. Influenced by other factors such as other runners’ performance while passing by, and magnified by extrinsic factors such as public cheers and behavior, and air temperature and humidity [25,26,27,29,37,38], they may have difficulties in assessing the situation, continuing to run instead of quitting the race, and collapse [29,35,36].

Endurance races are still associated with morbidity and mortality [4,39,40,41,42]. Although the absolute risk of sudden death for the participants in an endurance race is low (0.5–1.5 cases/100,000 runners), the tragedy itself is magnified due to the involvement of younger runners [3,4,5]. In a study published in 2012, the median age among deaths was 41.5 years. Half of the deaths (14/28) occurred in runners under the age of 45. The most common cause of death was cardiac arrest due to inherited/congenital heart disease [40]. The main reasons for death among runners older than 45 years (93%) was myocardial infarction (n = 13) and atherosclerotic heart disease (n = 14). In our study, over 40% of participants had high blood pressure with or without medication, and an additional 25% had Cerebrovascular Disease (CVD). This constitutes a significant risk factor and higher morbidity and mortality rate since history of CVD together with other possible risk factors such as chronic diseases, chronic prescription medication, and history of collapse during a race, have all proven to be alarming signals. Consequently, the idea of risk assessment and pre-participations’ screening has emerged to capture runners view on their health condition [43,44].

Runners’ views on their symptoms and their medical history are important factors in preventing medical encounters in endurance races [29,45]. In one study, the authors evaluated the efficacy and feasibility of an online pre-race medical screening and an educational intervention program to reduce medical complications in long-distance races. They found that all medical encounters (including life-threatening encounters) were significantly lower after the introduction of the program, which also was feasible to perform [45]. Educational interventions do not necessarily need to deal with medical information. Knowledge about other factors such as the impacts of diseases, personal characteristics at risk, as presented in our study may be part of intervention programs in order to reduce medical complications. Existing and hereditary diseases, and unexpected medical conditions, found in our study, despite its size, are in accordance with earlier reports and confirm the need for a pre-race screening test before endurance contests.

It is shown that with an increasing air temperature above an optimum, performance decreases. The optimal air temperature can vary between runners due to the level of physical readiness. Higher air temperature has shown to be associated with a higher rate of runners withdrawing from the race and increased medical complications. The acceptable upper limit for many races is based on WBGT (wet bulb globe temperature) of 28 °C; however, the WBGT is an empirically-based index, which does not provide any detailed examination of the meteorological variables contributing to thermal stress and is consequently of limited diagnostic value [21,22].

### Limitations 

One main limitation of this study was the low response rate in the questionnaire study, which was due to wrong or incomplete information. We have, however, been informed that registered runners can offer their start positions to other participants if they cannot take part in the race due to sickness or other private reasons. This change should be reported to the organizers at least 48 hours before the contest. Missing this mandatory task may result in names with no address or addresses with no names; an organizational problem that should be addressed.

Nevertheless, as an effect of the low response rate to the questionnaire and the qualitative characteristics of the study, no statistically significant data can be reported. There is a need for larger number of participants, ideally compared to a control group to determine the relative importance of a range of risk factors. The study, however, opens up new research fields and confirms the need for both quantitative and qualitative studies in this field.

Qualitative studies have the shortcoming of not being able to generalize to a population, but the results may be transferable and can provide researchers with new angles in front of a quantitative study. Considering the scale of the studied half-marathon (45,000 competitors and six years of data but only 164 runners picked up by ambulance due to collapse) a quantitative study will be extensive. It may benefit from tentative results provided by studies such as this one to verify or falsify 

## 5. Conclusions

Collapsing might be the first sign of severe medical conditions and clinically mimic the more common and benign exercise-associated collapse. Several risk factors associated with severe collapses have been reported. However, other individual and mental characteristics may indicate a unique profile for runners who collapse. Pre-race health check-ups and educational initiatives aiming at mental evaluation, preparedness and information before endurance races might be a necessary step to avoid life threatening complications.

## Figures and Tables

**Table 1:** Shows the frequency and percent of hereditary diseases in the investigated population.

Hereditary		
Hereditary Diseases	Frequency	Percent
High blood pressure with or without medication	7	41.2
Early myocardial infarction or stroke (<60 years of age) and other heart diseases	3 + 1	23.5
Systematic diseases (Myelofibrosis, SLE, MS)	1 + 1 + 1	17.7
Diabetes	1	5.9
Pulmonary diseases, including asthma	1	5.9
Regular medication for other diseases	1	5.9
Total	17	100.0

## Supplementary Materials

The following are available online at https://www.mdpi.com/2075-4663/8/1/2/s1, Swedish questionnaire.
