# Supplementary material for: Profiling Collapsing Half Marathon Runners—Emerging Risk Factors: Results from Gothenburg Half Marathon
Source: Sports (Basel). 2019 Dec 25;8(1):2. doi: 10.3390/sports8010002 (PMC7022577; doi:10.3390/sports8010002)
Supplement: Supplementary file 1 [file sports-08-00002-s001.pdf]

Enkät varvsforskning

Information till deltagare

Årligen genomförs över 800 långloppstävlingar i världen. Göteborgsvarvet är en av de största. Varje sådan tävling medför så kallade värmekollapser som behöver medicinsk tillsyn. Vi är en forskargrupp från Göteborgs och Karlstad universitet som intresserat sig för Göteborgsvarvet och medicinska säkerhetsfrågor i samband med tävlingen.

Detta är en enkät som vänder sig till dig som har transporterats med ambulans i samband med Göteborgsvarvet under åren 2010–2017. Syftet med enkäten är att få mer kunskap om orsaker till medicinska händelser och behov av ambulanssjukvård vid långlopp, så att sådana händelser i förlängningen kan förbyggas och hanteras på ett bättre sätt.

De omständigheter som bidrog till att du behövde och fick ambulanssjukvård kallas genomgående för ”händelsen” i de nedanstående frågorna.

Deltagandet är frivilligt och du kan när som helst avbryta, men dina svar är viktiga för forskningen och arbetet med att förbättra framtida arrangemang och råd till löpare. Studien är etikprövad. Alla svar behandlas konfidentiellt. Sammanställning och analys kommer enbart göras på aggregerad nivå. Det kommer inte framgå i projektets publikationer vem som svarat vad.

Har du några frågor gällande enkäten kan du kontakta Professor Eric Carlström på 070-273 81 26 alt. epost eric.carlstrom@gu.se

1. Tog du del av väderrapporten innan loppet (ringa in alternativ)?
   a. ja
   b. nej
   c. om ja, hur långt innan starten tog du del av väderrapporten? Antal timmar/minuter:
2. Vilket lufttemperaturintervall upplever du som mest behaglig att löpa i in allmänhet:
   mellan _____ °C och ______°C
3. Hur var du klädd under loppet? (ringa in alternativ)
   a. optimalt
   b. mindre optimalt
   c. om mindre optimalt, utveckla i vilket avseende (fritext):
4. Vilken dominerande färg hade din klädsel? (ringa in alternativ eller beskriv i fritext)
   a. mörk
   b. ljus
5. Vilken ungefärlig sluttid siktade du på?
6. Beskriv kort vad du åt och drack innan starten? Fritext!

A. <u>Hälsa</u>

7. Har du någon av följande tillstånd (ringa in alternativ)
   a. Högt blodtryck/medicinering för högt blodtryck
   b. Diabetes
   c. Höga blodfetter/medicinering för höga blodfetter
   d. Hjärta/kärlsjukdom
   e. Njursjukdom
   f. Lungsjukdom inkl. astma
   g. Regelbunden medicinering för någon annan sjukdom
   h. Annat som du tycker är viktigt (fritext):
8. Har dina föräldrar eller syskon någon av följande tillstånd (ringa in alternativ)
   a. Högt blodtryck/medicinering för högt blodtryck
   b. Diabetes
   c. Höga blodfetter/medicinering för höga blodfetter
   d. Tidig hjärtinfarkt el stroke (<60 års ålder)
   e. Annan hjärtsjukdom
   f. Plötslig död
   g. Njursjukdom
   h. Lungsjukdom inkl. astma
   i. Regelbunden medicinering för någon annan sjukdom
   j. annat som du tycker är viktigt (fritext):

B. <u>Allmänna uppgifter</u>

9. Ålder: ______ år
10. Kön (ringa in alternativ)
    a. man
    b. kvinna
    c. Annat
11. Vilken är din högsta utbildning (ringa in alternativ)
    a. grundskola
    b. gymnasium
    c. högskola/universitet
12. Yrke (fritext):

C. <u>Bakgrund/förberedelser</u>

13. Hur många långlopp liknande Göteborgsvarvet (d.v.s. minst 21km) hade du genomfört under en femårsperiod innan händelsen? _____
    Ange årtal och antal lopp
14. Hur långt sprang Du i genomsnitt i veckan året innan händelsen?
    Antal kilometer:
15. Hur långt sprang Du i genomsnitt i veckan 3–4 veckor före händelsen?
    Antal kilometer:
16. Fanns det omständigheter som påverkade din uppladdning för loppet negativt (sista månaden)? (ringa in alternativ)
    a. Ja
    b. Nej
    c. Om ja, beskriv omständigheterna (fritext)
17. Upplevde du att du hade en övertro på din förmåga att genomföra loppet (ringa in alternativ)?
    a. ja
    b. nej
18. Studerade du höjdkartan innan loppet (ringa in alternativ)?
    a. ja
    b. nej
19. Var det första gången du sprang Göteborgsvarvet? (ringa in alternativ)
    a. Ja
    b. Nej
    c. Om nej, vad var din ungefärliga sluttid gången före?

Om ja, provsprang du banan innan du deltog första gången (ringa in alternativ)

   d. ja
   e. nej

20. Hur ofta har du svårigheter med att avsluta de sista detaljerna i en uppgift/ett projekt när de mer krävande momenten är avklarade?
    a. Aldrig
    b. Sällan
    c. Ibland
    d. Ofta
    e. Mycket ofta
21. Hur ofta har du svårigheter med att få ordning på saker och ting när du ska utföra en uppgift som kräver organisation?
    a. Aldrig
    b. Sällan
    c. Ibland
    d. Ofta
    e. Mycket ofta
22. Hur ofta har du problem med att k omma ihåg avtalade möten, t.ex. läkarbesök eller åtaganden?
    a. Aldrig
    b. Sällan
    c. Ibland
    d. Ofta
    e. Mycket ofta
23. Hur ofta händer det att du undviker eller skjuter på att sätta igång med en uppgift som kräver mycket tankemöda?
    a. Aldrig
    b. Sällan
    c. Ibland
    d. Ofta
    e. Mycket ofta
24. Hur ofta händer det att du sitter och plockar med något, eller skruvar på dig ch rör händer eller fötter när du är tvungen att sitta stilla en längre stund?
    a. Aldrig
    b. Sällan
    c. Ibland
    d. Ofta
    e. Mycket ofta
25. Hur ofta händer det att du känner dig överaktiv och tvungen att hålla igång, som om du gick på högvarv.
    a. Aldrig
    b. Sällan
    c. Ibland
    d. Ofta
    e. Mycket ofta

26. Har du kollapsat i samband med fysisk aktivitet (ringa in alternativ)
    a. nej
    b. ja, vid träning
    c. ja, vid en tävling
    d. om ja, kommentar (fritext):
27. Hur var din hälsa de sista tre veckorna innan händelsen (ringa in alternativ)
    a. inga hälsoproblem
    b. hälsoproblem
    c. om hälsoproblem , beskriv närmare t.ex. bröstsmärta, svimning, eller hjärtklappning (fritext):
28. Hur ofta drack du alkohol året innan händelsen? (ringa in alternativ)
    a. aldrig
    b. några gånger per år
    c. någon gång per månad
    d. en gång i veckan
    e. flera gånger i veckan
29. Hur många glas drack du i genomsnitt en vanlig gång du drack alkohol innan händelsen (med ett glas menas 1 glas vin/ 1 starköl/4 cl starksprit, ange 0 om du inte dricker alkohol). Antal glas:

D. <u>Loppet</u>

30. Utifrån din önskade sluttid, hur låg du till i loppet? (ringa in alternativ)
    a. Snabbare än planerat
    b. Som planerat
    c. Långsammare än planerat
31. Drack du vid de vätskestationer du passerade innan händelsen (ringa in alternativ)?
    a. nej
    b. ja

E. Händelsen

Nedan har Du en karta över banan.

32. Markera följande på bifogad karta.
    a. Var skedde händelsen? **Markera med ett kryss.**
    b. Var blev du hämtad med ambulans? **Markera med en kvadrat.**
    c. Var började det bli tungt, det vill säga tilltagande trötthet/besvär som föregick händelsen? **Markera med en ring.**

33. Vilka var, enligt din egen uppfattning, de tre viktigaste faktorerna till händelsen (fritext)?

1: 2: 3:

34. Vilka av följande **inre faktorer** upplevde du minuterna/minuten innan händelsen (ringa in ett eller flera alternativ)

a. stress
b. inre krav
c. nervositet
d. hunger
e. törst
f. trötthet
g. kramper
h. yrsel
i. känsla av förvirring
j. illamående
k. synrubbning
l. huvudvärk
m. konstiga tankar
n. annat (fritext)

35. Vilka av följande **yttre faktorer** upplevde du minuten innan händelsen (ringa ett eller flera alternativ)

a. publik/publikens beteende
b. trängsel
c. anhöriga
d. hejarop
e. uppförslut
f. ojämnheter i gatan
g. värme
h. kyla
i. motvind
j. nederbörd
k. annat

36. I efterhand, anser du att du kunde ha förhindrat händelsen? (fritext):

F. Efterförlopp

37. När du brutit loppet, i väntan på ambulans, fick du någon typ av hjälp där du befann dig? (ringa in alternativ)

a. nej
b. ja
c. om ja, vilken typ av hjälp (fritext):
d. om ja, vem hjälpte dig (exempelvis åskådare, löpare, annan)? (fritext):
e. vet inte

38. Blev du inlagd på vårdavdelning? (ringa in alternativ)

a. nej
b. ja
c. om ja, **ange antal vårddygn:**

39. Blev du sjukskriven efter händelsen? (ringa in alternativ)

a. nej
b. ja
c. om ja, **ange dagar du var sjukskriven**: ……………..

40. Har du fortsatt springa efter händelsen? (ringa in alternativ)

a. ja
b. nej

41. Har du deltagit i några långlopp (mer än 21km) efter händelsen? (ringa in alternativ)

a. ja
b. nej

Övergripande kommentarer, ytterligare upplysningar (fritext):

G. Framtida kontakt

42. Får vi kontakta dig för uppföljande frågor? (ringa in alternativ)

a. ja
b. nej

Information för forskargruppen:

Varvstudie

Inklusion: Ambulanshämtningar kollaps el motsvarande, 144 fall perioden 2010-16, samt 2017
Kontrollgrupp*:* 300 fördelat på startgrupper under åren 2010-2017
Pilotstudie*, inklusion:* 15 ambulanshämtningar och 30 kontroll 2010-2017.
